# Seabuckthorn Reverses High-Fat-Diet-Induced Obesity and Enhances Fat Browning via Activation of AMPK/SIRT1 Pathway

**DOI:** 10.3390/nu14142903

**Published:** 2022-07-15

**Authors:** Yu Wang, Xuyang Gao, Xiaoyou Chen, Qiang Li, Xinrui Li, Junxing Zhao

**Affiliations:** College of Animal Science, Shanxi Agricultural University, Jinzhong 030801, China; wangyu20000602@163.com (Y.W.); xuyang2019@yeah.net (X.G.); chenxiaoyou0915@163.com (X.C.); lq994186@163.com (Q.L.); lxr9972@163.com (X.L.)

**Keywords:** seabuckthorn, obesity, brown adipose tissue, beige, AMPK, SIRT1

## Abstract

Seabuckthorn possesses various bioactive compounds and exhibits several positive pharmacological activities. The present trial aims to determine the effect of seabuckthorn powder intake on high-fat diet (HFD)-induced obesity prevention in mice. The results suggest that seabuckthorn powder intake decreased body weight, fat mass, and circulating lipid levels, and improved insulin sensitivity in HFD-fed mice. Moreover, dietary seabuckthorn powder alleviated hepatic steatosis and hepatic lipid accumulation induced by the HFD. Furthermore, seabuckthorn exhibited obvious anti-inflammatory capacity in white adipose tissue (WAT) by regulating the abundance of inflammation-related cytokines, such as interleukins 4, 6, and 10; tumor necrosis factor α; and interferon-γ. More importantly, dietary seabuckthorn powder promoted a thermogenic program in BAT and induced beige adipocyte formation in iWAT in HFD-fed mice. Interestingly, we found that seabuckthorn powder effectively restored AMPK and SIRT1 activities in both BAT and iWAT in HFD-fed mice. Collectively, these results potentiate the application of seabuckthorn powder as a nutritional intervention strategy to prevent obesity and related metabolic diseases by promoting thermogenesis in BAT and improving beige adipocyte formation in WAT.

## 1. Introduction

The rate of obesity is increasing rapidly throughout the world [1]. Mechanistically, obesity is attributed to the excessive growth and expansion of adipose tissue, and, to date, two types of adipose tissue have been identified, named white adipose tissue (WAT) and brown adipose tissue (BAT). In addition, a type of inducible adipocytes, named beige adipocytes, have been discovered within WAT. BAT possesses an energy-dissipating capacity, which declines in overweight or obese men [2], whereas the activation of BAT is a potential approach to combat obesity [3]. Similarly, obesity impairs beige adipogenesis by downregulating the expression of uncoupling protein 1 (UCP-1) [4]. Therefore, the activation of BAT and beige adipocyte function could be a feasible therapeutic strategy for obesity and type 2 diabetes mellitus prevention [5].

Seabuckthorn (*Hippophae rhamnoides* L.) is extensively distributed in Asia, Europe, and Canada, and has received worldwide attention for therapeutic, nutraceutical, and cosmetic purposes [6]. Owing to the presence of bioactive compounds, seabuckthorn extracts exhibit several positive pharmacological activities, including immunomodulatory and anti-aging, anti-oxidative, and anti-cancer activities, as well as lowering plasma cholesterol and improving lipid metabolism enzyme activity [7]. A previous study in mice proved that seabuckthorn extract prevents high-fat diet (HFD)-induced obesity, which it may do through regulating the key gene expression involved in both adipogenesis and lipogenesis [8]. Moreover, seabuckthorn extract ameliorates hepatic steatosis, insulin resistance, and inflammatory response [9]. However, the underlying mechanism of seabuckthorn extract on obesity disorder prevention is not clear.

AMP-activated protein kinase (AMPK) serves as a gauge of multiple metabolic pathways [10], which potentiate AMPK as a target for intervention in childhood and adolescent obesity [11]. Interestingly, a recent study reported that seabuckthorn fruit oil extract attenuates fat accumulation and improves lipid metabolism in hamsters, which is associated with AMPK activation [12]. Moreover, AMPK activity is indispensable for brown and beige adipogenesis [13], implying that dietary seabuckthorn may attenuate HFD-induced obesity through the modulation of BAT and beige adipocyte function. Therefore, the purpose of the current trial was to study the role of dietary seabuckthorn in HFD-induced obesity prevention. We hypothesized that seabuckthorn intake would ameliorate HFD-induced obesity and insulin resistance through the modulation of AMPK activity and BAT/beige function.

## 2. Materials and Methods

### 2.1. Care and Use of Animals

Twenty-four male mice (C57BL/6J, eight weeks old) were divided into three groups and fed ad libitum with a normal diet (chow group; D12450B, SPF Biotechnology Co., Ltd., Beijing, China), an HFD (HFD group; 60% energy from fat, D12492, SPF Biotechnology Co., Ltd.), and an HFD containing 0.3% (3 g/kg) seabuckthorn freeze-dried powder (HFDSB group; Xi’an Changyue Biological Technology, Co., Ltd., Xi’an, China). The powder was obtained by freeze-drying whole seabuckthorn berries and contains all parts of the seabuckthorn berry, including the seeds, pulp, and peel. The animals were individually housed in cages under a 12:12 h light/dark cycle.

### 2.2. Glucose and Insulin Tolerance Tests

For the glucose tolerance tests (GTT), the mice were moved into clean cages at 5:00 p.m. (24 h before the test) and fasted for 16 h with drinking water as normally supplied. The mice were injected with D-glucose (2 g/kg body weight). The blood glucose of mice was monitored at 0, 30, 60, 90, and 120 min after injection. For the insulin tolerance tests (ITT), the mice were fasted for 4 h and followed by intraperitoneal injection with human insulin (1 U/kg body weight). Blood glucose was measured at 0, 30, 60, 90, and 120 min after injection. The glucose concentrations from the tail veins were measured using a glucometer (Sinocare Inc. Co., Ltd., Changsha, China).

### 2.3. Cold-Induced Thermogenesis Test

A cold-induced thermogenesis test was conducted at the 12th week of the experiment. The mice were moved to clean cages and deprived of food and water. Then, the mice were subjected to cold stimulation (4 °C, 6 h). Thermal images before and after cold stimulation were captured using a camera (FLIR E6xt, FLIR Systems OÜ, Tallinn, Estonia).

### 2.4. Hematoxylin and Eosin Staining

Adipose tissue and liver samples fixed by 4% paraformaldehyde solution were dehydrated by passing samples sequentially through 70%, 80%, 90%, 95%, and 100% alcohol and xylene. After wax infiltration, the specimens were paraffin-embedded and sectioned with a microtome (RM2265, Leica, Wetzlar, Germany). Subsequently, the dewaxed and rehydrated sections were stained with hematoxylin and eosin (H&E) as previous described [14]. For each sample, 10 randomly selected fields were captured using a microscope (DMi8, Leica, Wetzlar, Germany), and the diameter of the adipocytes was measured using ImageJ software (NIH, Bethesda, MD, USA).

### 2.5. Immunohistochemistry

Immunohistochemistry was performed in deparaffinized sections prepared as described above. For antigen retrieval, the sections were incubated in the citrate buffer (pH 6.0, 100 °C) for 20 min. The sections were cooled down and rinsed in PBS for 3 times. Sections were blocked using 1% BSA in TBS for 2 h at 37 °C, followed by incubation with UCP1 antibody (overnight, 1:50). Samples were treated with 0.3% H_2_O_2_ for 15 min at room temperature and incubated with biotinylated goat anti-rabbit IgG and streptavidin–peroxidase complexes (SA1022, Boster Bio-engineering Co., Ltd., Wuhan, China). Finally, sections were treated with 3,3′-diaminobenzidine tetrahydrochloride (DAB) for color development (AR1022, Boster Bio-engineering Co., Ltd., Wuhan, China) and counterstained with hematoxylin. Images were captured using a Leica DMi8 microscope.

### 2.6. Enzyme-Linked Immunosorbent Assay

The frozen liver samples were powdered in a mortar containing liquid nitrogen, and the powder (1 g) was homogenized in ice-cold ethanol (9 mL, 100%). Then, the homogenates were centrifuged for 10 min at 2500× rpm at 4 °C, and supernatants were used for ELISA. High-density lipoprotein cholesterol (HDL-C, A113-1-1), low-density lipoprotein cholesterol (LDL-C, A112-1-1), cholesterol (TC, A111-1-1), and triacylglycerol (TG, A110-1-1) abundances in the liver and serum were measured using their corresponding kits (Nanjing Jiancheng Bioengineering Institute, Nanjing, China) following the manufacturer’s manual. Serum TNF-α (MU30030), IL-6 (MU30055), and IL-10 (MU30044) were determined using ELISA kits from Bio-Swamp Co., Ltd. (Wuhan, China).

### 2.7. Western Blotting

Western blotting was performed using the previous protocol from our laboratory with minor revisions [15]. Briefly, the mouse BAT and WAT samples were cooled using liquid nitrogen and ground in a mortar. The tissue powder was homogenized in lysis buffer. After centrifugation, the supernatant was collected and used for protein separation in SDS-PAGE (4 °C, 100 V for 2 h). The isolated proteins were transferred to nitrocellulose membranes at 100 V, 4 °C for 1.5 h. The membranes were blocked with 5% nonfat dry milk in PBS and sequentially subjected to primary (4 °C, overnight) and secondary antibody (room temperature, 1 h) incubation. Staining was developed using the Odyssey Infrared Imaging System (LI-COR Biosciences, Lincoln, NE, USA). The relative expression of proteins was normalized to β-actin content.

### 2.8. Quantitative Real-Time PCR (qPCR) Analysis

Total RNAs were extracted using the TRIzol reagent (Sigma, Saint Louis, MO, USA) following the manufacturer’s instructions. The qualities of RNAs were monitored using a NanoDrop instrument (ND-2000, Thermo Scientific, Rockfork, IL, USA). Samples with 260/280 ≥ 1.9 were used for the following steps. The cDNAs were synthesized using PrimeScript RT kits (Takara, Dalian, China), and qPCR was performed using SYBR Green fluorescent dye (Takara, Dalian, China) in a CFX Connect Real-time PCR Detection System (Bio-Rad, Hercules, CA, USA). The reaction steps were as follows: 95 °C for 30 s, 95 °C for 20 s, 60 °C for 30 s, 40 cycles. β-tubulin was used as the housekeeping gene. The 2^−ΔΔCT^ method was applied for the relative analysis of target gene expression. The primer sequences used in the qPCR are listed in Table 1.

### 2.9. Antibodies

The antibodies against PR domain-containing 16 (PRDM16, bs-19986R), UCP1 (bs-1925R), NAD-dependent deacetylase sirtuin-1 (SIRT1, bs-2257R), AMPKα1 (bs-1115R), pAMPKα1 (bs-5551R), peroxisome proliferator-activated receptor γ coactivator 1α (PGC-1α, bs-1832R), and β-actin (bsm-33036M) were obtained from Biosynthesis Biotechnology Co., Ltd. (Beijing, China). Goat anti-rabbit secondary antibody (926-32211) and anti-mouse secondary antibody (926-68070) were obtained from LI-COR Biosciences (Lincoln, NE, USA).

### 2.10. Statistical Analysis

Statistical analysis was performed using GraphPad Prism 9 (Monrovia, CA, USA). One-way ANOVA was performed to analyze the mean differences among groups, and Tukey’s honestly significant difference test (Turkey’s HSD) was used for pairwise mean comparison. Data are presented as mean ± SEM; *p* < 0.05 was considered as statistically significant.

## 3. Results

### 3.1. Seabuckthorn Inhibits Weight Gain and Adiposity of Mice Fed with HFD

**Figure 1:**
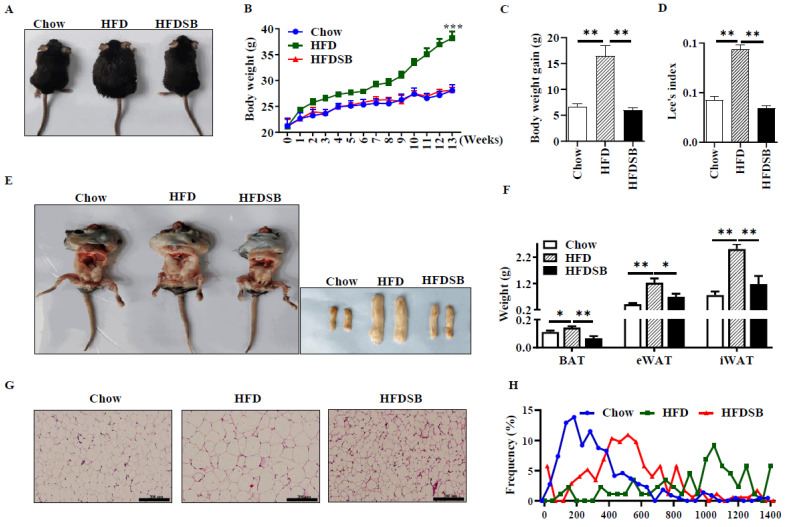
Effects of seabuckthorn intake on weight gain and adiposity of mice. (**A**) Representative images of body size. (**B**) Weekly body weight. (**C**) Body weight gain. (**D**) Lee’s index. (**E**) Anatomical image for intra-abdominal fat (left) and white adipose depots (right). (**F**) Weight of BAT, eWAT, and iWAT. (**G**) H&E staining of WAT sections (scale = 200 µm). (**H**) Distribution of white adipocyte size. (Mean ± SEM; *n* = 8 in each group; *, *p* < 0.05, **, *p* < 0.01, ***, *p* < 0.001).

Mice in the HFD group exhibited greater weight than those in the Chow group, which was recovered by seabuckthorn supplementation (Figure 1A–C, *p* < 0.01). Consistently, Lee’s index was reduced in the HFDSB group compared with the HFD group (Figure 1D, *p* < 0.01). The mice fed with the HFD exhibited an enlarged accumulation of intra-abdominal fat, while seabuckthorn intake remarkably attenuated this effect. Moreover, although the HFD increased the weights of BAT, eWAT, and iWAT, those in the HFDSB groups approached the average weight of chow-fed mice (Figure 1E,F, *p* < 0.01). In addition, there were no differences for food intake among the three groups (data not shown). Furthermore, H&E staining indicated that the seabuckthorn-fed mice had a smaller adipocytes size in iWAT (Figure 1G,H).

### 3.2. Seabuckthorn Intake Improved Insulin Sensitivity and Glucose Tolerance

**Figure 2:**
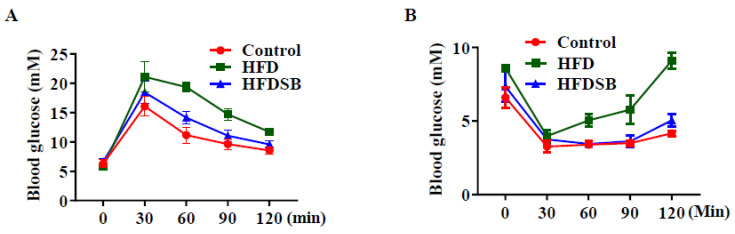
Glucose tolerance test (GTT) and insulin tolerance test (ITT) in mice fed with different diets. (**A**) Blood glucose levels following GTT test. (**B**) Blood glucose levels following ITT test. HFD, high-fat diet; HFDSB, high-fat diet containing 0.3% seabuckthorn freeze-dried powder (mean ± SEM; *n* = 8 in each group).

Compared with the HFD group, the seabuckthorn-fed mice displayed a better tolerance to glucose load during the glucose tolerance test (Figure 2A). Furthermore, the mice in the HFDSB group vs. those in the HFD group showed increased insulin sensitivity during the insulin tolerance test (Figure 2B).

### 3.3. Seabuckthorn Prevented Hepatic Steatosis Induced by HFD

**Figure 3:**
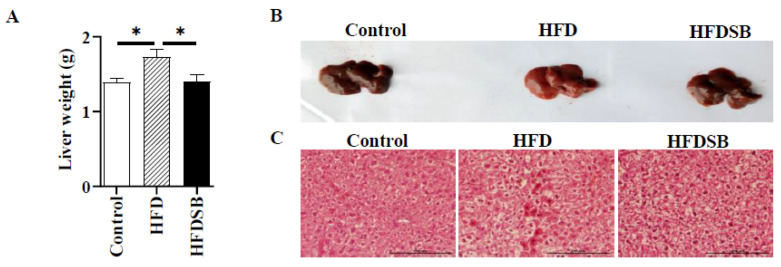
Seabuckthorn prevented hepatic steatosis induced by HFD. (**A**) Liver weight. (**B**) Images of liver in different group. (**C**) Representative H&E staining for liver sections (scale = 200 μm; mean ± SEM; *n* = 8 in each group; * *p* < 0.05).

HFD consumption led to a heavier liver, and seabuckthorn powder intake effectively reversed such effect (Figure 3A). Morphology and H&E staining suggested that HFD consumption induced abnormal triglyceride accumulation in the liver, while minimal lipid deposition was observed in the livers of the HFDSB-fed mice (Figure 3B,C). As expected, feeding mice with an HFD for 13 weeks resulted in hyperlipidemia, which was evidenced by reduced HDL-C and elevated LDL-C, TG, and TC in both the serum and liver (Table 2). Compared with the HFD-fed mice, the hyperlipidemia was markedly alleviated in the seabuckthorn-fed mice (Table 2).

### 3.4. Seabuckthorn Attenuates Inflammation in HFD-Fed Mice

**Figure 4:**
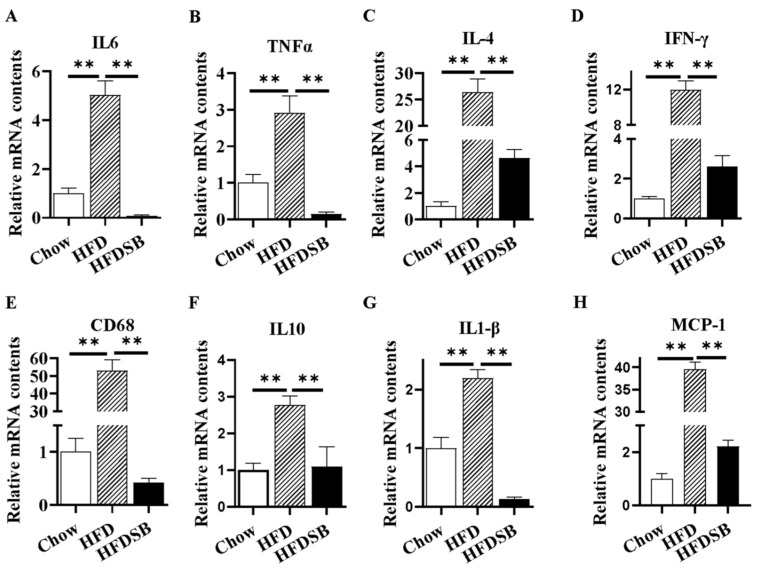
Inflammatory-related gene mRNA expression in different groups in inguinal white adipose tissue (iWAT). (**A**–**H**) stand for mRNA contents of *interleukin 6* (*IL-6*), *tumor necrosis factor α* (*TNF-α*), *interleukin 4* (*IL-4*), interferon-γ (*IFN*-γ), CD 68, *interleukin 10* (*IL-10*), *interleukin β* (*IL-β*), and *monocyte chemoattractant protein-1* (*MCP-1*) in iWAT, respectively (mean ± SEM; *n* = 8 in each group; ** *p* < 0.01).

The ELISA results revealed that Ll-6, IL-10, and TNF-α were elevated in the serum from the mice fed with the HFD, while seabuckthorn intake significantly decreased HFD-induced inflammation (Table 3). In agreement, the mRNA expression levels of inflammation-related markers in iWAT, including *TNF-α*, *MCP-1*, *CD68*, *IL-4*, *IL-6*, *IL-10*, *IL-β*, and *IFN-γ*, were altered among the groups (Figure 4A–H, *p* < 0.01), suggesting that seabuckthorn alleviates adipose tissue inflammation by regulating inflammatory gene expression.

### 3.5. Seabuckthorn Enhanced the BAT Function in HFD-Fed Mice

**Figure 5:**
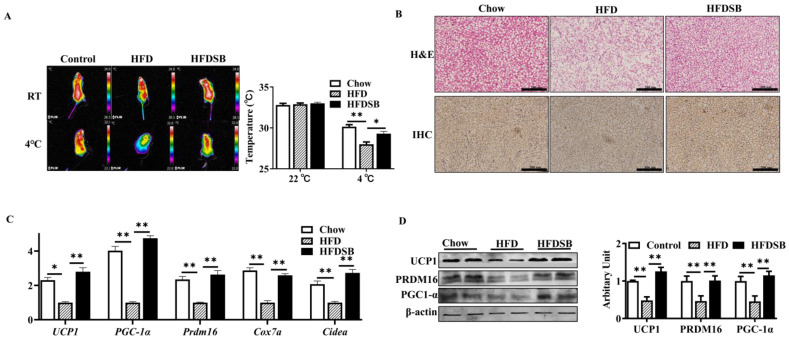
Seabuckthorn intake regulated the function of brown adipose tissue (BAT). (**A**) Representative thermal images. (**B**) H&E (upper) and immunohistochemical (lower, anti-UCP1) staining of BAT sections from different groups. (**C**) mRNA levels of *Prdm16*, *Pgc1α*, *Ucp1*, *Cox7a*, and *Cidea* in BAT. (**D**) Protein contents of PRDM16, PGC-1 α, and UCP1 in BAT (scale: 200 µm; mean ± SEM; *n* = 8 in each group; ** *p* < 0.01 and * *p* < 0.05).

As Figure 5A shows, no body surface temperature differences were observed among the different groups before cold exposure. Compared with the chow group, the mice fed with the HFD showed a lower surface temperature after cold stimulation (*p* < 0.01), and seabuckthorn intake rescued this decline (*p* < 0.05). Compared with the control mice, the BAT from the HFD-fed mice exhibited an obvious phenotype that resembled white fat, while dietary seabuckthorn remarkably reduced lipid accumulation in BAT (Figure 5B). Furthermore, the downregulated mRNA abundances of *Prdm16*, *Ucp1*, *Pgc-1α*, *Cox7a*, and *Cidea* in the BAT of the HFD-fed mice were recovered by the intake of seabuckthorn powder (Figure 5C, *p* < 0.01). In agreement, the inhibitory effects of an HDF on PRDM16, UCP, and PGC-1α protein abundances were alleviated by dietary seabuckthorn (Figure 5D, *p* < 0.01).

### 3.6. Dietary Seabuckthorn Supplementation Potentiated Beige Adipogenesis

**Figure 6:**
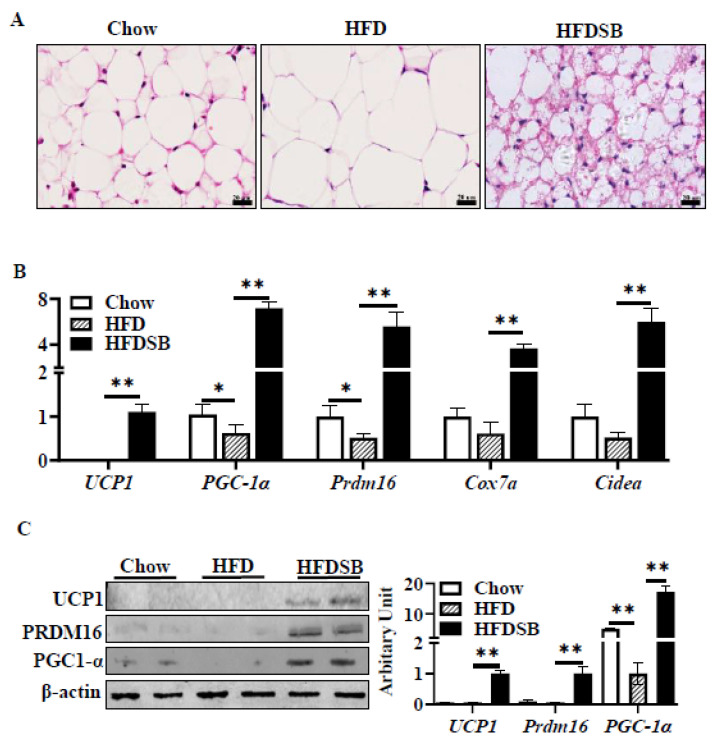
Dietary seabuckthorn intake induced beige adipogenesis in iWAT. (**A**) H&E staining of iWAT sections. (**B**) mRNA expression of *Prdm16*, *Pgc1α*, *Ucp1*, *Cox7a*, and *Cidea* in iWAT. (**C**) Protein abundances of PRDM16, PGC-1 α, and UCP1 in iWAT (scale: 20 µm; mean ± SEM; *n* = 8 in each group; ** *p* < 0.01 and * *p* < 0.05).

To determine whether seabuckthorn could promote beige adipogenesis in iWAT, a histological analysis was performed. We found that more multilocular beige adipocytes were accumulated in the iWAT of the HFDSB group (Figure 6A). Moreover, the thermogenic gene expressions were greater in the iWAT of the seabuckthorn-fed mice (Figure 6B), indicating that an enhanced formation of beige adipocytes occurred. Consistently, the alteration of key protein contents, including UCP1, PRDM16, and PGC-1α, further confirmed more beige adipocyte formation in the iWAT of the HFDSB group.

### 3.7. Effects of Seabuckthorn on AMPK/SIRT1 Activity in Both BAT and iWAT

**Figure 7:**
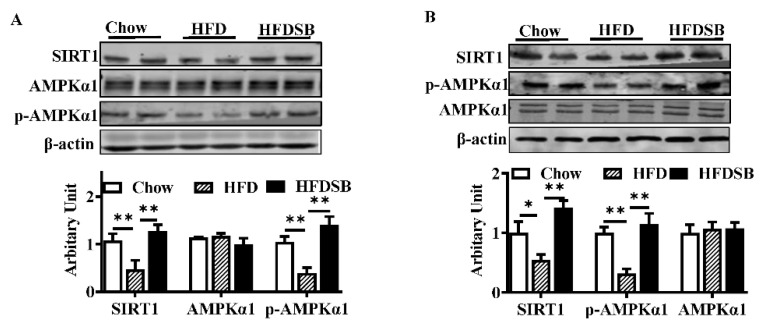
AMPKα1 and SIRT1 protein abundances in different groups. (**A**) Protein contents of SIRT1, AMPKα1, and phosphorylated AMPKα1 in BAT. (**B**) Protein abundances of SIRT1, AMPKα1, and phosphorylated AMPKα1 in iWAT (mean ± SEM; *n* = 8 in each group; * *p* < 0.05, ** *p* < 0.01).

Reduced AMPKα1 protein abundances in both BAT (Figure 7A) and iWAT (Figure 7B) from the HFD mice were observed, while seabuckthorn effectively alleviated the inhibition. In addition, SIRT1 protein contents were also recovered in both the BAT (Figure 7A) and iWAT (Figure 7B) of the HFDSB group.

## 4. Discussion

Obesity has placed a huge burden on the global health-care system [16]. Approximately 60% of the US population is recognized as overweight or obese [17], while about half of all adults and a fifth of all children are considered overweight or obese in China [18]. Although appropriate lifestyle intervention is crucial to weight loss success, maintenance is extremely challenging. To date, pharmacological strategies have been developed for obesity treatment, such as the use of sibutramine, fluoxetine, sertraline, orlistat, and topiramate [19]. However, these medicines should be used with caution because of the side effects and potential for drug abuse [20]. On the other hand, studies have investigated the potential application of herbal medicine and natural products for treating diet-induced obesity [21]. Despite previous studies suggesting the anti-obesity function of seabuckthorn extracts in animal models [22], the use of seabuckthorn itself is limited. Indeed, seabuckthorn freeze-dried powder retains almost all its useful nutrients, including those found in the fruit oil, pulp, and peel. Considering the fact that seabuckthorn is usually utilized as a whole fruit, a study using the whole fruit would be more meaningful.

In the present trial, compared with the control mice, the mice in the HFD group gained a greater body weight, whereases seabuckthorn powder consumption recovered the increased body weight gain. Moreover, the consumption of seabuckthorn powder effectively alleviated HFD-induced adiposity, as evidenced by elevated TC, TG, and LDL-C levels. Therefore, the alteration of body weight in the HFDSB group could be at least partially a result of the lower body fat accumulation. In line with our results, previous reports indicated that the ethanolic extract of seabuckthorn leaves [8], flavonoid glycosides extracted from seabuckthorn leaves [9], and seabuckthorn polysaccharide [23] prevent HFD-induced obesity in mice. Given that an ideal natural anti-obesity product may only be considered effective if it reduces the initial body weight by 10% [24], these results strengthened the proposals regarding the health effects of seabuckthorn powder on obesity and lipid metabolism disorders induced by an HFD.

Obesity is always associated with type 2 diabetes with the induction of systemic insulin resistance. Both the GTT and ITT results confirmed that the HFD induced insulin resistance in mice, whereas dietary seabuckthorn freeze-dried powder effectively accelerated the rates of blood glucose clearance, indicating that certain bioactive compounds in seabuckthorn could be used as an insulin sensitizer, which may be at least partially attributed to the high concentration of omega-3 fatty acids [25]. Similarly, seabuckthorn fruit oil extract has been proved to attenuate insulin resistance via the activation of the PI3K/Akt pathway [26]. Systemic insulin resistance promotes nonalcoholic fatty liver disease progression, which, in turn, exacerbates insulin action and eventually forms a vicious cycle [27]. In the present study, along with improved insulin sensitivity, hepatic steatosis induced by the HFD was ameliorated in seabuckthorn freeze-dried powder-fed mice. Fatty acid β-oxidation in mitochondria is a catabolic process by which fatty acids are broken down. A previous study indicated that bioactive compounds, such as polyphenols, polysaccharides, and flavonoids, enhance the β-oxidation of fatty acids [28], and, therefore, such bioactive compounds in seabuckthorn freeze-dried powder might contribute to hepatic steatosis recovery.

Not only is adipose tissue a major metabolic organ that is responsible for systemic energy, glucose, and lipid homeostasis, but it also functions as an endocrine organ [29]. Indeed, WAT is recognized as a primary tissue where obesity-related inflammation is triggered and exacerbated. In an obese state, macrophages within adipose tissue are polarized into pro-inflammatory M1 macrophages, causing pro-inflammatory cytokine secretion, and, subsequently, this contributes to local and systemic inflammation and insulin resistance [30]. Here, we found that abundances of inflammatory markers induced by HFD consumption in WAT were dramatically decreased in the mice fed with seabuckthorn freeze-dried powder, which contributed to the noticeable improvement in insulin sensitivity and adiposity.

As mentioned above, despite studies proving the anti-obesity function of seabuckthorn extracts, the underlying mechanism remains undefined. Given that both BAT and beige can effectively dissipate energy as heat and contribute to energy expenditure, stimulating BAT activity and/or the browning of WAT have emerged as worthy strategies to combat obesity [31,32]. Therefore, we asked if seabuckthorn freeze-dried powder could enhance BAT activity and beige adipocyte formation. We found that an obvious morphological change in BAT was observed in the HFDSB group, which contained considerably smaller lipid droplets than that in the HFD-fed mice. Moreover, brown adipogenesis potential was restored, which was confirmed by the upregulation of PRDM16, a key transcription coregulator controlling brown adipocyte formation. Noticeably, mice in the HFDSB group exhibited a markedly improved thermogenesis capacity, together with a greater expression of thermogenic genes. In addition to BAT, beige adipocytes also have a thermogenic and energy-dissipating function due to the existence of mitochondrial UCP1. A previous study documented that inflammation of WAT is accompanied with diminished generation of beige adipocytes [4]. Given that brown and beige adipocytes have many distinguishing characteristics [33], the possible effects of seabuckthorn powder on beige adipogenesis were also evaluated. In the current study, dietary seabuckthorn powder consumption caused a decrease in the adipocyte size in WAT, and more importantly, obvious beige adipocytes with the appearance of multilocular lipid droplets within iWAT were observed, which were further confirmed by dramatically upregulated UCP1. These results potentiate the browning effects of seabuckthorn on iWAT.

Finally, we explored the potential mechanisms by which seabuckthorn powder regulated brown and beige adipogenesis. It is clear that AMPK activity is necessary for the normal differentiation of many cell types [34]. Particularly, AMPK activity is required for the browning of WAT and maintaining BAT function by epigenetically regulating *Prdm16* transcription [35,36], Here, we found that both BAT and iWAT in the HFDSB groups showed elevated AMPK activity, which further contributed to greater *prdm16* transcription, resulting in the upregulation of genes involved in BAT/beige adipocyte function. SIRT1 serves as an NAD-dependent deacetylase and exerts various biological functions, including glucose and lipid metabolism [37]. Moreover, SIRT1 stimulates both the differentiation and activation of BAT [38], as well as the formation of beige adipocytes via the deacetylation of PPARγ [39]. Here, we observed that SIRT 1 contents were increased in both BAT and iWAT in the HFDSB group, which is in accordance with a previous report indicating that seabuckthorn seed protein enhances the activity of the AMPK/SIRT1 pathway [40]. Reciprocally, AMPK functions as an SIRT1 activator by increasing the cellular NAD^+^, while SIRT1 improves AMPK activity via the deacetylation and activation of liver kinase B1 (LKB1, upstream kinase of AMPK) [41]. Therefore, the enhanced BAT function and beige adipogenesis in mice fed with seabuckthorn powder might be attributed to the activation of AMPK/SIRT1.

## 5. Conclusions

We observed that seabuckthorn powder reversed HFD-induced obesity, improved systemic insulin sensitivity, and promoted thermogenic program in BAT and WAT, which might be attributed to the activation of the AMPK/SIRT1 pathway. Our results potentiate the application of seabuckthorn as a nutritional intervention strategy to prevent obesity and related metabolic diseases.

## Figures and Tables

**Table 1:** Primer sequences for quantitative real-time PCR.

Gene	Forward	Reverse
*Ucp1*	ACTGCCACACCTCCAGTCATT	CTTTGCCTCACTCAGGATTGG
*Prdm16*	CTCGAATGGACAAACGGCCT	GGTACCCTGGCTTTGGACTC
*Cox7α*	CAGCGTCATGGTCAGTCTGT	AGAAAACCGTGTGGCAGAGA
*Cidea*	ATCACAACTGGCCTGGTTACG	TACTACCCGGTGTCCATTTCT
*Pgc-1α*	TGCAGCGGTCTTAGCACTC	GAGGAGTTAGGCCTGCAGTT
*CD68*	TGTCTGATCTTGCTAGGACCG	GAGAGTAACGGCCTTTTTGTGA
*TNF-α*	GCCAACGGCATGGATCTCAA	TAGCAAATCGGCTGACGGTG
*IL-1β*	TCGCAGCAGCACATCAACAA	TCCACGGGAAAGACACAGGT
*IL-6*	CCACTTCACAAGTCGGAGGC	TCTGCAAGTGCATCATCGTTGT
*IL-4*	AGTGAGCTCGTCTGTAGGGC	CAGGCATCGAAAAGCCCGAA
*IL-10*	TGGGTTGCCAAGCCTTATCG	TCAGCTTCTCACCCAGGGAA
*IFN-γ*	CTTCAGCAACAGCAAGGCGA	CATTGAATGCTTGGCGCTGG
*MCP-1*	CCCCAAGAAGGAATGGGTCC	GGTTGTGGAAAAGGTAGTGG
*β-tubulin*	TGCCAGGATTAGCACCCTTG	TCGAACACCTGTTGGGTCAG

**Table 2:** Hepatic and plasma TC, TG, HDL-C, and LDL-C levels among groups.

Catalog	Parameter	Chow	HFD	HFDSB
Serum	TG (mM)/L	1.06 ± 0.12	1.26 ± 0.10 ^#^	0.59 ± 0.05 **
	TC (mM)/L	5.32 ± 0.62	6.97 ± 1.10 ^#^	4.46 ± 0.29 **
	HDL (mM)/L	3.22 ± 0.24	2.87 ± 0.08 ^#^	3.30 ± 0.03 *
	LDL (mM)/L	0.94 ± 0.13	1.95 ± 0.63 ^#^	1.04 ± 0.29 **
Liver	TG (mM/g)	1.00 ± 0.11	2.90 ± 0.17 ^#^	1.33 ± 0.17 *
	TC (mM/g)	1.00 ± 0.02	1.58 ± 0.06 ^#^	0.65 ± 0.03 **
	HDL (mM/g)	1.92 ± 0.14	1.26 ± 0.02 ^#^	1.00 ± 0.10 *
	LDL (mM/g)	1.00 ± 0.01	1.27 ± 0.11 ^#^	0.63 ± 0.01 *

^#^ *p* < 0.05, compared between control and HFD-fed mice; * *p* < 0.05, ** *p* < 0.01, compared between HFD and HFDSB groups. LDL, low-density lipoprotein cholesterol; HDL, high-density lipoprotein cholesterol; TG, triacylglycerol; TC, cholesterol. Data are expressed as the means ± S.E.M.

**Table 3:** Serum levels of IL-6, IL-10, and TNF-α.

Parameter	Chow	HFD	HFDSB
IL-6 (pg/mL)	9.82 ± 2.31	48.82 ± 4.72 ^##^	12.45 ± 3.08 **
IL-10 (pg/mL)	35.33 ± 3.03	48.25 ± 4.68 ^##^	19.29 ± 2.53 **
TNF-α (pg/mL)	18.31 ± 2.56	60.19 ± 5.48 ^##^	37.88 ± 2.75 **

^##^ *p* < 0.01, compared between control and HFD-fed mice; ** *p* < 0.01, compared between HFD and HFDSB. IL 6, interleukin 6; IL 10, interleukin 6; TNF-α, tumor necrosis factor α.

## Data Availability

The data presented in this study are available upon request from the corresponding author.
